# Enhancing participation to health screening campaigns by group interactions

**DOI:** 10.1038/srep09904

**Published:** 2015-04-23

**Authors:** Raffaella Burioni, Pierluigi Contucci, Micaela Fedele, Cecilia Vernia, Alessandro Vezzani

**Affiliations:** 1 Dipartimento di Fisica e Scienza della Terra, Università di Parma and INFN, Gruppo collegato di Parma; 2 Dipartimento di Matematica, Università di Bologna; 3 Dipartimento di Scienze Fisiche Informatiche e Matematiche, Università di Modena e Reggio Emilia; 4 CNR, Istituto Nanoscienze S3, Modena

## Abstract

Improving the prevention efficacy of health screening campaigns by increasing their attendance rate represents a challenge that calls for new strategies. This paper analyzes the response to a Pap test screening campaign of 155,000 women over the last decade. Using a mathematical model of statistical physics origins we derive a quantitative estimate of the mutual influence between participating groups. Different scenarios and possible actions are studied from the cost-benefit perspective. The performance of alternative strategies to improve participation are forecasted and compared. The results show that the standard strategies with incentives concentrated toward the low participating groups are outperformed by those toward pivotal groups with higher influence power. Our method provides a flexible tool useful to support policy maker decisions while complying with ethical regulations on privacy and confidentiality.

Screening campaigns are usually devised within health systems to detect anticipatory signs of serious, life threatening, illnesses by preliminary tests with the purpose to prevent them or deal with them at early, non-lethal, stages^1^,^2^,^3^. Their overall efficacy depends both on a wide adhesion of the screenable population^4^ and on a statistically fair participation of all the different social groups involved. Low participation rates within some groups (*non-responders*) are a challenge to policy makers still waiting for a solution^5^,^6^.

The participation promotion to a screening test is most commonly realized by individual invitations (by letters, by voice of the general practitioner, etc.) to the candidates, supported in some cases by education and awareness programs. Those methods succeed to raise the attendance up to some point^7^, but have recently proven to be quite useless to increase it further.

The enormous development of the information technology suggests that a possible improvement could be obtained analyzing the huge electronic archives of health data collected in the last two decades. The challenge of Big Data approach on healthcare is to extract the maximum desired information from collective anonymous data while fully respecting privacy and confidentiality within ethical regulations^8^,^9^,^10^,^11^,^12^,^13^,^14^,^15^. In this paper we present a possible approach to improve screening campaigns that fulfills those requirements and has the ability to infer a minimal model to make useful predictions.

In particular, we study participation data to the Papanicolaou smear (Pap) test, a screening test used to prevent cervical cancer by detecting potentially pre-cancerous and cancerous processes in the women endocervical canal. The campaign, following the EU recommendations^16^, should cover 95–98% of the target population and reach an attendance of 60% or higher to be successful.

The choice of each woman to participate in the screening campaign is related both to her individual attitude to the invitation and to peer-to-peer effects, arising from the interaction with other women involved in the campaign^17^. Some of them are deeply aware of the importance of the test and will consequently participate. Others will not for several reasons: not understanding the purpose of a Pap test, costs, attitudes and beliefs about cancer (fatalism, etc.) and logistical factors (transportation, childcare, etc.)^6^,^18^,^19^. Moreover, women in minority groups, women with low incomes or education levels and women not sexually active are less likely to enter the screening programs^20^. Most part of the population does not have a strong personal opinion about the Pap test and will likely be influenced by the other women’s advices and choices^21^. Even though the mix of the individual attitude and peer-to-peer mechanisms in leading to the final decision differs from woman to woman, the analysis of the empirical data about the screening campaign allowed us to retrace some similarities, in particular among individuals of the same generation.

We approach the problem of improving the attendance to the test from a novel perspective, based on ideas, data analysis techniques and mathematical methods borrowed from statistical physics. Recently, similar approaches have been applied successfully to shed the light on different social phenomena related to health and the quality of life^22^,^23^.

Our main innovative feature is the introduction, measurement and control of the peer-to-peer (interaction) effects typical of the social behavior^24^,^25^,^26^,^27^, which are not taken into account in the standard discrete choice approach^28^,^29^,^30^. The inference of the model parameters in discrete choice is based on the measure of mean values of the attendance, while fluctuations are merely used as error estimates. Conversely, our approach relies intrinsically on the measure of fluctuations and correlations to infer the set of parameters, that include interactions. Since in the typical screening program each woman is invited only few times (typically once every 2–5 years), fluctuations and correlations between single individuals cannot be effectively measured. The huge dimension of the dataset, covering a large part of the population, allows instead for a precise estimate of the correlations between groups. In this perspective, the natural approach is a multi-populated mean field model^31^.

As a case study, we analyze data from the campaign suggested by the Regional Health System and conducted in the district of Parma, in Northern Italy, from 2004 to 2012 on an average annual population of 120.000 women (see Methods for a detailed data analysis). Through the campaign, all women aged 25–64 (target population) in the district of Parma were invited to have a free Pap test every three years, by sending an invitation letter^32^ and a reminder after 2–4 months, if the individual does not respond to the first invitation. The choice of the case study was mostly motivated by the database richness and the rigorous care it has been crafted with. From this extensive dataset, we determine the free parameters of a mathematical model describing the probability distribution of the participation by measuring the average, the fluctuations and the correlations of the attendance in the three age groups that naturally arise from the data analysis: young, middle and senior women. The introduction of the interaction parameters is strongly motivated by the fact that the observed fluctuations and correlations are significantly larger than those typically produced by mutually independent random variables.

Once the free parameters are computed from real data, the mathematical model describing the probability distribution of choices is fully operative to forecast the system behavior when these parameters are changed. We analyzed several strategies to increase the global participation as well as the participation of the youngest group, which turns out to be the less respondent to classical invitations. The strategy targeting only the less-responders produces very modest results on the overall attendance, while a strategy targeting the pivotal middle age group and increasing the strength of their interaction with the other groups has definitely better performances.

Our method represents a flexible tool to enhance participation in presence of robust historical data. Its predictive ability may be used to help and assist policy makers decisions.

## Results

### Interaction effects from data analysis

**Figure 1:**
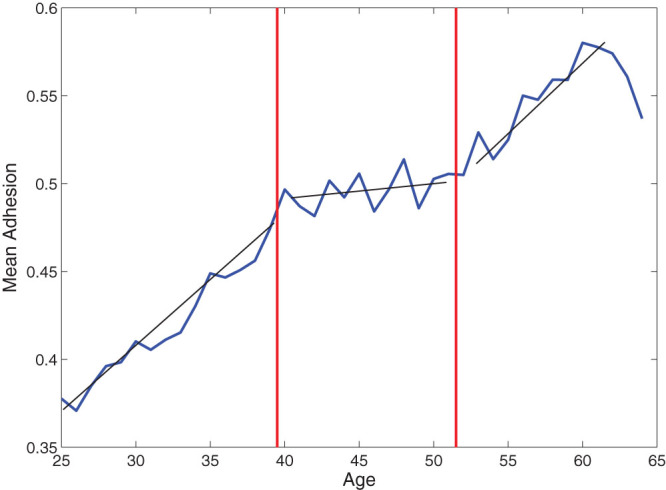
Mean adhesion to the screening program on first invitation, averaged over the whole time period 2004–2012, as a function of age of the target population.

**Figure 2:**
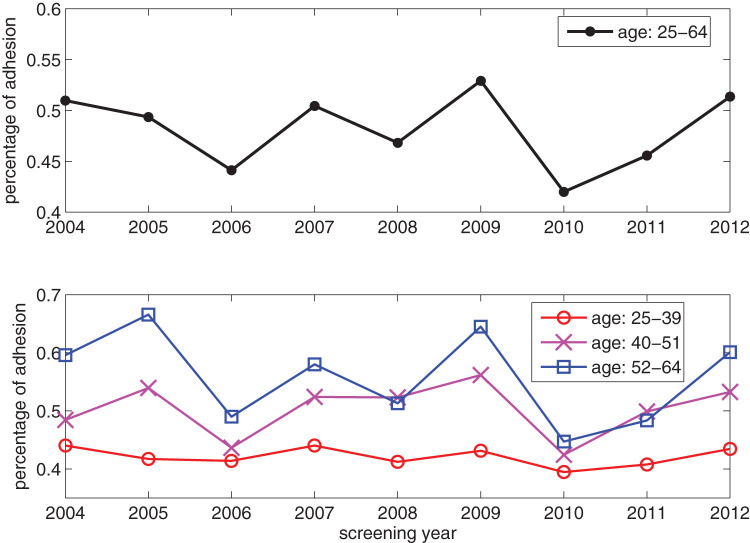
Percentage of adhesion to screening on first invitation as a function of the screening year. Upper Panel: Percentage of participation to first screening invitation for the entire target population (25–64 years old). Lower panel: Percentage of participation to first screening invitation for the youngest group (25–39 years old, red circles), for the middle group (40–51 years old, magenta crosses) and for the oldest group (52–64 years old, blue squares).

**Figure 3:**
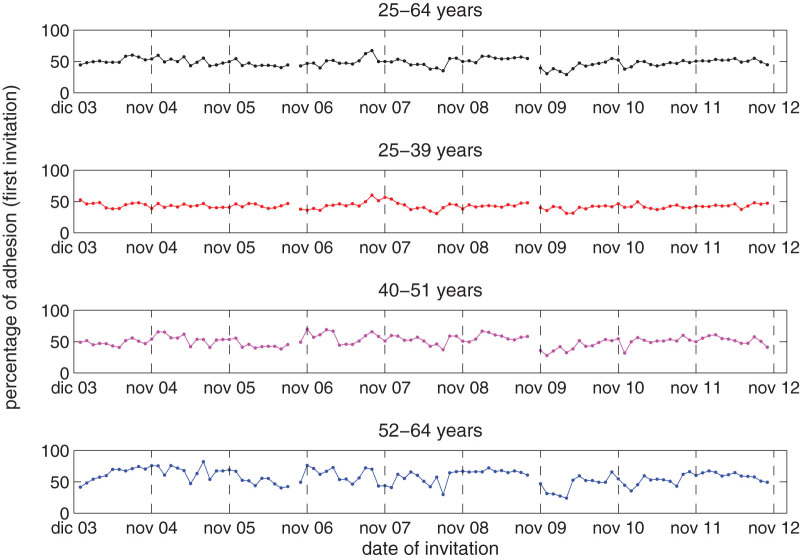
Percentage of adhesion to screening invitation as a function of the date of the first invitation, for the entire dataset (first panel) and for the three age groups (last three panels).

To test the relative factors involved in the individual choice and the role of peer-to-peer effects, we analyzed our dataset to create a suitable partition of the women involved in the campaign. The data analysis of the available attributes pointed out that age is the main discriminant in attendance behavior. Figure 1 displays the adhesion rate to the first invitation versus the woman age averaged over the whole time period and shows that the set of women is naturally divided into three age groups: from 25 to 39, from 40 to 51 and from 52 to 64. In each group this rate grows linearly (apart from small oscillations) but at different speed. Moreover, the average adhesion in the three age sectors features a coherent behavior during all the examined screening period (9 years, see Fig. 2). Interestingly, the two age classes separators coincide with two meaningful age thresholds in women life statistics. In fact, 39 is the age at which 90% of women with children had their first child, with a distribution displaying a very sharp decrease at that age. 52 is the average age of menopause in Italy (data from ISTAT 2011). Therefore, these thresholds can be associated to significant changes in women’s social environment and attitude towards the screening campaign. According to this finding, we consider three groups: *G*_1_ = {women from 25 to 39 years old}, *G*_2_ = {women from 40 to 51 years old} and *G*_3_ = {women from 52 to 64 years old} and we build a three-populated mean-field model to describe their decisions to attend the Pap test (see Methods for details). The percentage of adhesion for the whole dataset and for the three age groups is represented in Fig. 3. In this context such a model^31^,^33^,^34^,^35^ is the simplest probabilistic description that associates to each age group the probability of adhesion to the test that depends on individual factors as well as on mutual interactions.

Denoting with *N* the number of women involved in the campaign we codify their individual choices with the dichotomous variables:
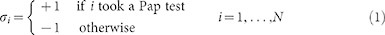
and their average choice with the variable:
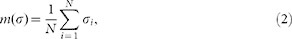
so that, denoted by *p* the global attendance to the test, the following relation holds 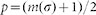. The joint probability distribution of the choices of all the women, 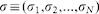 is described by
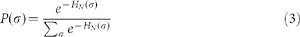
where −*H_N_* is an utility function embedding our a priori knowledge of the choice mechanism.

We first assume that a woman has an inclination strength to attend the screening test according to the group she belongs to. We identify those inclinations with three parameters 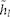 with *l = * 1, 2, 3 denoting the age groups. Moreover, the peer-to-peer interaction affecting the choice is ruled by six couplings among the groups: three of them for inter-group interactions, and the other three for intra-group interactions. In particular we denote by 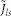 for *l, s = * 1, 2, 3 the parameter that tunes the interaction between a woman of the group *G_l_* and one of the group *G_s_* (assumed to be symmetric). These assumptions lead to the following mean-field function *H_N_*:
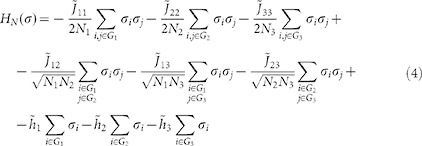
where *N_l_* with *l* = 1, 2, 3 is the size of the group *G_l_*.

The strategy we pursue is to derive the value of the nine parameters from the experimental data through the so-called *inverse problem*^36^,^37^,^38^,^39^. For an exactly solvable model, as the one we consider, this can be efficiently achieved by expressing the model parameter as a function of the distributional moments (mean values, fluctuations and correlation) which can be estimated from the data set. In particular, denoted by *m_l_*(σ) the average choice of the group *G_l_*, by *m_l_* its expectation value in the large *N* limit and by 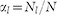 the relative size of the group *G_l_*, we can write the interaction matrix 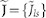 with *l*, *s = * 1, 2, 3 as
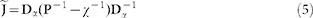
where 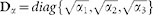, 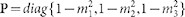, and χ is the matrix of elements
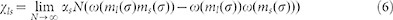
where 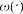 denotes the expectation value with respect to the measure (3). Once the matrix 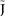 is determined, the parameters 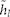 for *l* = 1, 2, 3 are obtained in the following way:
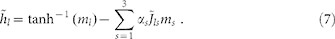
Estimated the average value and the correlations of the women’s average choice in the age groups from the data:
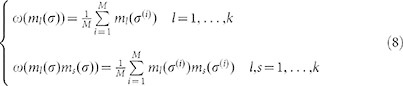
where 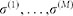, with *M* = 105 is a sample of independent configuration of choices (for further details on the inversion procedure and the sample of configurations see Methods), we obtain:
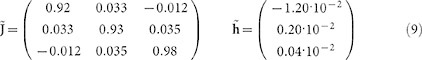


### Strategies to enhance participation

The previous results show that the three groups have a level of individual motivation to attend the screening test that grows with age. The high value of the self interactions 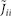 is a quantitative measurement of the coherence of behavior within each group. The non diagonal terms of the matrix 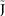, all of the same order of magnitude up to a factor 3, show the existence of a pivotal group, the middle age women, well connected to both the younger and the older women as expected for generational proximity. The pivotal role of the group *G*_2_ will be proposed to build an effective strategy of participation increase.

The standard incentive system to screening participation is to increase the individual availability, i.e. increase the parameters 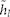 (individual inclinations). The invitation with a letter, the suggestion by the general practitioner and also the advertising on media belong to this type of actions. Acting on these parameters has a cost that is proportional to the number of people, and an unit cost per person that can be reasonably parametrized by
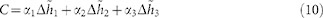
where *α_l_*, *l* = 1, 2, 3 is the relative size of each group on *N* = *N*_1_ *+ N*_2_ + *N*_3_ and 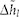 is the variation of the *l*-th parameter.

**Figure 4:**
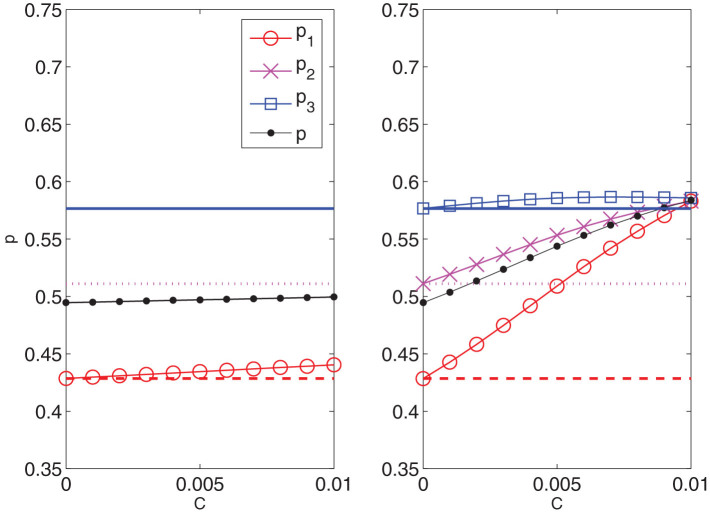
Participation *p*_1_, *p*_2_, *p*_3_ and *p* of the three age groups and of the total population as a function of the cost *C*. Left panel: Adhesion forecast provided by the model without interaction. The cost *C* varying in 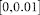 is obtained by changing the parameter *h*_1_ varying in 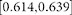. Right panel: Adhesion forecast provided by the model (4). The cost *C* is increased from 0 to 0.01 by changing the parameter 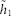 varying in 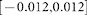 (the rest of the entries of the matrices 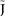 and 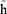 (see eq. (9)) are unchanged). For all panels the horizontal lines represent the initial conditions of the improvement strategies. Dashed red lines refer to group *G*_1_, dotted magenta lines to the group *G*_2_ and continuous blue lines to *G*_3_. Red circles, magenta crosses and blue squares measure the participation of the first, second and third group, respectively, as the cost of the campaigns varies. The global adhesion is represented by black dots.

We first proceed by comparing the forecasts provided by a model without interaction (standard discrete choice^28^), and a model which includes the interactions. Fig. 4 shows what are the effects, under the same unit cost, of increasing the individual incentive of the less responders, i.e. the young women. One can see that in the case without interactions (left panel), the adhesion presents a very small increase only for the targeted group. Conversely, when the interaction is allowed, the participation of the same group increases from 50% to 58.4% (right panel). Moreover, the interacting case shows quantitatively the dragging effect of the group on the other two and suggests to exploit it to optimize the efficacy of the campaign.

**Figure 5:**
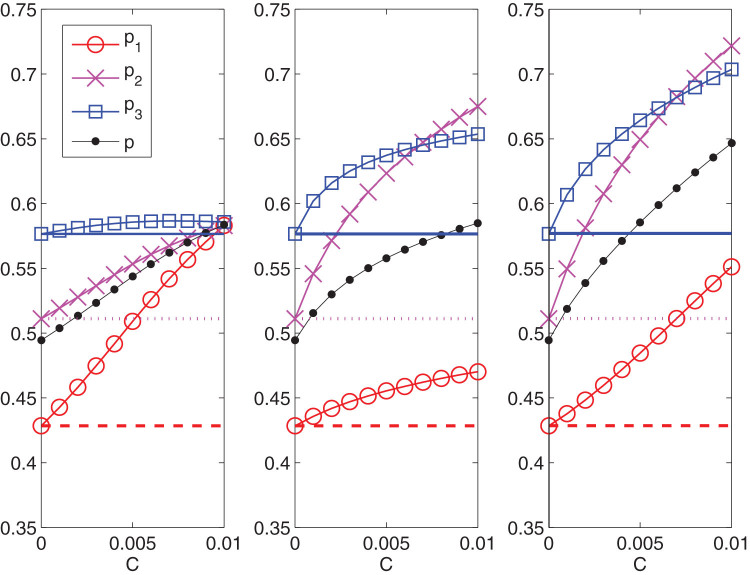
Participation vs. costs for three different strategies. Left panel: the whole cost is invested toward the lower participating group (group *G*_1_). Middle panel: the cost is invested toward the group with higher influence (group *G*_2_). Right panel: the cost is invested toward the group with higher influence together with an increase of a factor 2 of the interaction strength related to the same pivotal group *G*_2_, namely 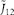 and 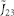. For all panels the dashed red line refers to the percentage of adhesion of the youngest group (25–39 years old), the dotted magenta line to the percentage of adhesion of the middle group (40–51 years old) and the continuous blu one to the percentage of adhesion of the oldest group (52–64 years old) as initial condition of the three improvement strategies. Red circles, magenta crosses and blue squares measure the participation of the first, the second and the third group, respectively, as the cost of the campaigns varies. The global adhesion is represented by black dots.

In Fig. 5 we proceed by comparing, still at a given cost, two new strategies to the previous interacting one: the first where we act on the middle age group by individual incentives (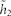) and the second where we couple the same action with an increased intensity of the two parameters 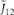 and 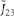, by a factor 2. Not only the third panel shows an increase in participation of the targeted group but it reveals an homogeneous increase of the other two groups leading to a substantial global result that crosses the bound of the 60% as recommended by the EU guidelines^16^.

**Figure 6:**
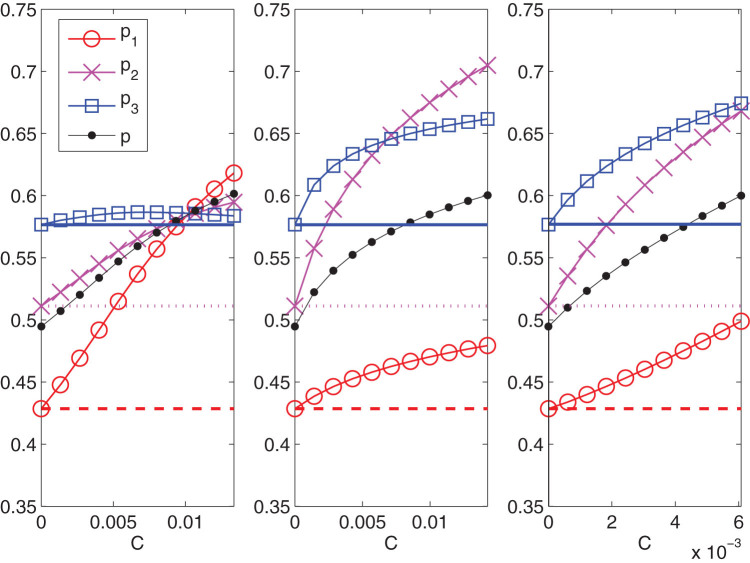
Same strategies of **Fig. 4 studied to reach a global 60% participation.** For the first and the second strategy the costs are doubled.

In order to convey to the policy makers the full capability of our method we also perform an analysis of the three strategies at fixed performance. For instance we set a fixed 60% global threshold and we analyze the different costs corresponding to different strategies (see Fig. 6). The first strategy (incentives directed only on group *G*_1_, the non responders) has a unit cost of 0.013, the second (incentives only on group *G*_2_) has a unit cost of 0.014 and the third (incentives on group *G*_2_ and increase of interactions) has a cost of 0.006. In other terms the last strategy comes with a saving of about 55% with respect to the first and the second one. The saved part can in turn be invested either to cover the costs, if any, of the increased interactions, or more likely to increase further the participation.

## Discussion

By studying a large database of participation response to a screening program for the Pap test, we have shown how to quantitatively estimate the peer-to-peer interaction among the relevant participating groups. We compare then two forecasted responses by varying the estimated parameters: the classical one, obtained by solely increasing the individual incentives, and the one where the incentives are coupled to interaction effects. We show that the second method is substantially more effective than the first not only in increasing global participation but, especially, in improving the participation of the non-responders.

The results have been achieved by describing the screening attendance with the help of a multi-populated mean-field model, with women divided in three groups by age. This approach is motivated by the fact that our dataset only allows for a collective investigation, as the choice of each woman is recorded only few times in the considered time period (9 years), and the partition by age was the most relevant one.

The entire analysis included within this work has been done in a range of parameters 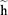 and 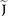 that does not come with abrupt swings. The considered model has indeed the possibility^40^,^41^ to display phase transitions. The investigation of those events is extremely interesting not only from the theoretical point of view but also for the possible applications. The system could indeed have desirable quick changes as well as disastrous ones. Both the study of the analytical solution as well as the inverse problem have to be carried on with different methods in that case and we plan to investigate this matter in future works.

The application of our method may reach far beyond the enhancement of screening campaign participation. In fact, it can be applied to forecast and improve all phenomena of preventive health and help policy makers to choose the best strategies.

## Methods

### Data description and statistical analysis

Our work is based on the analysis of participation data to a screening campaign for the prevention of cervical cancer, suggested by the Regional Health System and conducted in the district of Parma, in Northern Italy, from 2004 to 2012. The smallest geographical unit for which data are available is the administrative unit called “Municipality”. The district of Parma is organized in 47 municipalities, including the city of Parma.

Through the campaign, all women aged 25–64 (target population) in the district of Parma are invited to have a free Pap test every three years, by sending an invitation letter^32^. To enhance participation, if an individual does not respond to the first invitation, a second reminder letter is sent after 2–4 months. In our statistical analysis we consider only adhesion on the first invitation.

The female population resident in the considered area and aged between 25 to 64 years is composed by 119,302 women (102,778 italian and 16,524 foreign, data from ISTAT 2011). All municipalities, from the smallest (137 women aged 25–64 years out of 567 residents, males and females) to the largest one (50,927 women aged 25–64 years out of 175,895 residents, males and females) have been involved in the screening campaign.

For each woman, our dataset contains data relative to her age, the place of residence, the dates on which the invitations were sent, and those on which the test was planned and carried out. The data are automatically collected by the Parma Sanitary Unit. Due to privacy and ethical constraints, the huge dataset we investigate is formatted in anonymous form, so that only general information is available on the screened individuals. This limitations on the dataset only allow for average and mean field investigations, as the participation data cannot be related to specific persons. Conversely, the limitations preserve the right to the privacy in personal choices, that is very strict in health related subjects. In literature other strategies of investigation have been considered to measure the local structure of peer to peer effects in health choices^27^, and they necessarily call for informed consensus on small groups of people.

The dataset consists of 495,210 data entries over the period 2004–2012. In this period, three entire screening routines have been completed and each woman has been typically invited at least three times. The number of distinct women in the dataset is 163,272, but only 155,221 of them received at least one first invitation. In the considered period, 51,778 received at least three first invitations and 40,637 women received only one first call. These are typically women either aged near 64 in the first routine (that in the successive routine will go outside the screening program) or the youngest individuals (25 years old) that have been invited for the first time in the last three years routine. In this dataset we also find women that accede to the service without invitation: these are women either aged outside the target population (older than 65 years or younger than 25 and for this reason not included in the screening campaign) or that decide to take the test spontaneously (for example by paying a ticket for the health service). These women are not considered in our study, but this does not impoverish the statistics, since they correspond to the 9% of the total entries in the dataset.

Besides the data obtained from the screening campaign, we also consider data from an extended period of 10 years in the pre-screening regime, referring to the same municipalities, in an integrated form. In fact, prior to the screening campaign, there was the possibility to obtain the free Pap test. In this case, the typical spontaneous adhesion to the test procedure was of about 18% of the screenable population. This percentage was remarkably stable in time, did not depend on the age of the participants nor on different municipalities.

To test the relative factors involved in the individual choice and the role of peer-to-peer effect, we analyzed our dataset to create a suitable partition of the women involved in the project.

First, we observe that there are not substantial differences in the adhesion in each municipality, except for a few cases of small towns, where the different value of the participation percentage is affected by insufficient statistics and large fluctuations. Therefore, to enhance our statistics, we consider the aggregated data for the whole district of Parma.

On the contrary, age appears to be a relevant attribute for screening participation. By plotting the monthly adhesion separately for each age, from 25 to 64 years old women, we observe that adhesion typically increases with age. In particular, as shown in Fig. 1 women can be divided in three age groups: from 25 to 39, from 40 to 51 and from 52 to 64. In Fig. 2 we show the percentage of adhesion to screening on first invitation as a function of the screening year, in an aggregated form and in different age sectors. For example in 2005, the percentage of women screened within the program was 49.36%, but the youngest group (25–39 years) features a participation of 41.71%, the middle group (40–51 years) of 53.94% and the oldest one (52–64 years) of 66.56%.

In order to estimate from the data the averages and the fluctuations required for the inversion procedure we need to detect a set of independent realizations of the data. We consider, as independent statistical samples, the set of first invitations chosen automatically by the screening procedure, once a month from the set of women that have been invited, for the last time, later than three years before. This generates a set of 105 monthly samples of question/response of the invited women. The first measure is taken in December 2003, covering the invitations for January 2004, and the last measure refers to October 2012 (for the invitation of November 2012), see Fig. 3. The three breaking off points in the sample curves in Fig. 3 indicate the change from a screening routine to the successive (every three years there is a stop in sending invitation to work off the remaining queue of the round): the first round end in September 2006, the second in October 2009 and the third in November 2012. As the individuals to be invited each month are chosen randomly among the set of women invited more than three years before, our samples can be considered to be independent and representative of the whole sample with good accuracy. The number of women invited each month ranges from about 1,500 to 5,000, so that the statistical significance of each monthly dataset is high.

An important test has been performed on the robustness of the age partition. We have explained in fact that the choice of the three groups has been initially guided by the emerging average behavior shown in Figure 1. To verify the consistency of the group partition with the proposed model we have proceeded in several directions. First we have shown that by varying the age group thresholds the output matrix 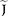 of (9) becomes progressively off-diagonal, i.e. the diagonal terms decrease and the off diagonal ones increase. An even stronger off-diagonalization is reached by performing a further subdivision of one or more classes. Finally, a completely off-diagonal matrix is obtained by choosing an arbitrary reshuffling of the individuals among the different classes.

To complete a consistency test on the proposed model, we have also computed the fourth order moments from the analytical solution and compared them with those directly derivable from the data. The result we have obtained is compatible with the errors predicted with the sample size that we used.

### Inversion of the model's exact solution and parameter evaluation from real data

Our approach is based on the introduction of a utility function^42^,^43^,^44^ embedding our a priori knowledge of the choice mechanism and the partition in groups. Our aim is to derive the best distribution from observed data with the least possible assumptions^45^,^46^.

Codifying an individual choice with the dichotomous variable 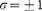 defined in (1) the utility function −*H_N_* (*H_N_* is called Hamiltonian in statistical mechanics, or cost function in applied fields) will in general depend on the choice of a population of *N* individuals 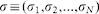. The probability distribution we consider is described by (3). We build the utility function in successive steps that refer to the temporal phases of screening. We also include a measure of the effect of the common cultural tendency toward the test through an inverse problem applied to a non interacting model, before the screening phase.

The idea is to first model the system in the pre-screening phase where the mean value of the attendance was stable in time and for different municipalities. Those data, collected before 1998, emerged from voluntary participation to the test without any invitation. The decision was taken primarily under the suggestion of the gynecologist, therefore without the influence of other women involved in the screening campaign. We model this by an Hamiltonian:
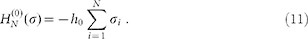


Borrowing terminology from magnetic spin systems we call *h*_0_ the pre-screening magnetic field that measures the cultural tendency toward the test and *m*(σ), the women's average choice to take the test defined in (2), magnetization. Since the pre-screening adhesion does not depend on age classes, we can assume that *h*_0_ is a global field. In the large N limit, the computation of the average value of the magnetization with respect to the Boltzmann-Gibbs distribution related to the function (11) is elementary and provides a relation between the observed average choice *m*_0_ and the field *h*_0_
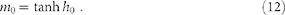


From a screening attendance frequency of 18%, corresponding to the value of *m*_0 _ = −0.64, we deduce:
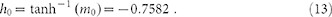


During the screening phase, we describe the peer-to-peer effect as a two-body term in the utility function. This modellization is suggested by the fact that the observed data display fluctuations that are interpretable as emerging from an interacting system. To start with we choose to model the interaction with a mean-field interaction with a unique population whose output will serve as a calibration of the interaction size. We also consider the appearance of a new field *h* possibly accounting for a shifted common individual tendency to take the test induced by the campaign effects. The cost function that describes the situation is:
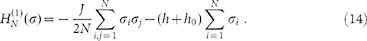


The procedure to estimate the global interaction parameter *J* and the global external field *h* starts from the observation that in the large *N* limit, the expectation of the magnetization 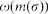 is equal to the stable solution of the following equation:
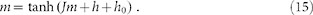


By differentiating this equation with respect to the external field we obtain the susceptibility χ:
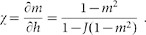


Therefore:
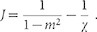


Since the partial derivative with respect to *h* of the expectation of the magnetization 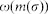 is:
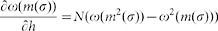
we have:
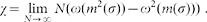


Thus:
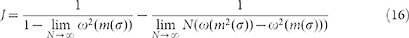
while the global external field *h* can be obtained by inverting the mean field equation (15):
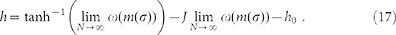
We calculate our estimates for the average value and the variance of the magnetization in the following way:
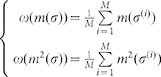
where 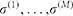 is a sample of independent and identically distributed configurations. In our case, the *M* sample configurations are the *M* = 105 monthly samples, extracted from the 9-years monthly invitations to the Pap test. From the data, we obtain the following values:
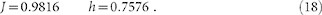
For the complete screening phase the cost function can be recasted as:
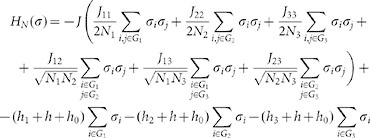
where *N_l_* for *l* = 1, 2, 3 is the number of women of the groups *G_l_*, *J_ls_* for *l*, *s* = 1, 2, 3 is the parameter that tunes the interaction between a woman of the group *G_l_* and one of the group *G_s_* (assumed to be symmetric), *h_l_* for *l* = 1, 2, 3 is the magnetic field acting on group *l* while *J*, *h* and *h*_0_ are those obtained in the previous step. Denoting by *m*_1_(σ), 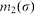 and *m*_3_(σ) respectively the magnetization of the groups *G*_1_, *G*_2_ and *G*_3_ and by *α_l_*, *l* = 1, 2, 3 the relative size of each group on 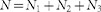 we can rewrite the cost function (19) as:
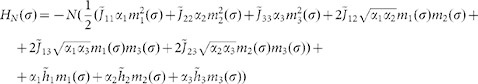
where 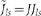 and 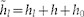 for 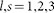. In the large N limit, the expectation of the magnetization on each group, 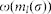, is equal to the value *m_l_* such that 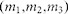 is a stable solution of the following system:
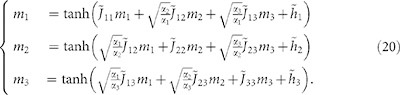
The elements of the susceptibility matrix χ, i.e. 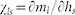, can be written as:
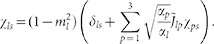
Therefore:
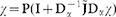
where 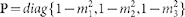, **I** is the identity matrix, 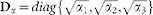 and
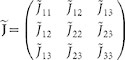
is the symmetric interaction matrix. Thus, 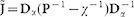 where the elements of the matrix χ can be computed using the following formula (6). Once the matrix 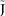 is determined, the elements 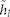 for 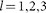 are obtained by inverting the mean field equations (20). Therefore, estimated the average value and the correlations of the magnetizations from the data as shown in (8) and extracted from the data the relative average sizes of each group, yielding:
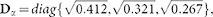
we obtain the parameters values shown in (9).

### Participation enhancement strategies

The three strategies that we propose to enhance participation to the screening campaign, (see Fig. 5), are obtained in the following way. We consider the solution 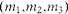 of the system (20) together with the total magnetization 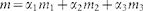 as a function of the cost *C* defined by (10) varying in [0, 0.01]. For the first strategy, acting on incentives for the less responding group, we choose to increase the cost *C* from 0 to 0.01 in eleven equally spaced steps, by changing only the magnetic field parameter 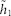 in [−0.012, 0.012]; for the second strategy, that focus on the middle age group, the increasing of *C* is obtained by changing only 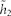 in the range [0.002, 0.033] at constant step; finally for the third strategy, we decide to increase the cost *C* from 0 with the same variation of 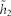 used in the second strategy and in addition we choose to change also the two values 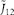 and 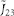 with linear laws in the intervals 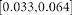, 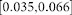, respectively, in order to double their initial values after eleven equally spaced steps (the rest of the entries of the matrices 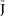 and 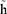 in eq. (9) being unchanged). In all panels of Fig. 5 the circles refer to the first group, the crosses to the second and the squares to the third one; the horizontal coloured lines correspond to the experimental values of the sample magnetizations 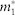 (dashed red line), 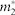 (dotted magenta line) and 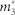 (continuous blue line) solution of the system (20) for 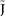 and 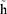 given by (9). Fig. 6 represents the cost for the three previous strategies payed to reach a global participation of 60%.

## Author Contributions

All the authors: R.B., P.C., M.F., C.V. and A.V. have equally contributed to each part of the scientific work presented in the paper.
